# α-Amino-3-hydroxy-5-methyl-4-isoxazole Propionic Acid (AMPA) and *N*-Methyl-d-aspartate (NMDA) Receptors Adopt Different Subunit Arrangements*

**DOI:** 10.1074/jbc.M113.469205

**Published:** 2013-06-11

**Authors:** Dilshan Balasuriya, Tom A. Goetze, Nelson P. Barrera, Andrew P. Stewart, Yuki Suzuki, J. Michael Edwardson

**Affiliations:** From the ‡ Department of Pharmacology, University of Cambridge, Tennis Court Road, Cambridge CB2 1PD, United Kingdom,; the § Departamento de Fisiología, Facultad de Ciencias Biológicas, Pontificia Universidad Católica de Chile, Alameda 340, Santiago, Chile, and; the ¶ Department of Chemistry, Graduate School of Science, Kyoto University, Kitashirakawa-oiwakecho, Sakyo-ku, Kyoto 606-8502, Japan

**Keywords:** Atomic Force Microscopy, Cell Surface Receptor, Glutamate Receptors, Ionotropic (AMPA, NMDA), Protein Complexes, Single-particle Analysis

## Abstract

Ionotropic glutamate receptors are widely distributed in the central nervous system and play a major role in excitatory synaptic transmission. All three ionotropic glutamate subfamilies (*i.e.* AMPA-type, kainate-type, and NMDA-type) assemble as tetramers of four homologous subunits. There is good evidence that both heteromeric AMPA and kainate receptors have a 2:2 subunit stoichiometry and an alternating subunit arrangement. Recent studies based on presumed structural homology have indicated that NMDA receptors adopt the same arrangement. Here, we use atomic force microscopy imaging of receptor-antibody complexes to show that whereas the GluA1/GluA2 AMPA receptor assembles with an alternating (*i.e.* 1/2/1/2) subunit arrangement, the GluN1/GluN2A NMDA receptor adopts an adjacent (*i.e.* 1/1/2/2) arrangement. We conclude that the two types of ionotropic glutamate receptor are built in different ways from their constituent subunits. This surprising finding necessitates a reassessment of the assembly of these important receptors.

## Teaser

**Background:** Recent evidence has suggested that the various ionotropic glutamate receptors are assembled in the same way from their constituent subunits.

**Results:** Atomic force microscopy imaging of antibody-decorated AMPA and NMDA receptors revealed different subunit arrangements.

**Conclusion:** AMPA receptors have an alternating subunit arrangement, whereas NMDA receptors adopt an adjacent arrangement.

**Significance:** Our results necessitate a reassessment of ionotropic glutamate receptor assembly.

## Introduction

Ionotropic glutamate receptors mediate fast excitatory neurotransmission in the vertebrate brain and are critical for normal brain function and development (1, 2). Dysfunction of these receptors has been implicated in numerous neurological and psychiatric disorders, such as chronic pain, Alzheimer disease, and schizophrenia (1).

All three ionotropic glutamate receptor subfamilies (*i.e.* AMPA-type, kainate-type, and NMDA-type) assemble as tetramers of four homologous subunits. The various subunits share a common modular architecture, consisting of an extracellular N-terminal domain (NTD),^4^ an agonist-binding domain (ABD), a transmembrane domain (TMD), and an intracellular C-terminal domain (CTD; 1, 2). AMPA and kainate receptors can function as homomers, although *in vivo* they preferentially assemble as heteromers. In contrast, NMDA receptors are obligate heteromers usually composed of two GluN1 and two GluN2 subunits. Heteromeric AMPA and kainate receptors appear to have a 2:2 subunit stoichiometry and an alternating subunit arrangement (3, 4). However, there have been conflicting results regarding the subunit arrangement in NMDA receptors, with evidence for either an adjacent (5, 6) or an alternating arrangement (7–10).

We have developed a method, based on AFM imaging, for determining the arrangement of subunits within ionotropic receptors (11–13). The method involves engineering specific epitope tags onto each subunit and expressing the receptors in a suitable cell line (tsA 201). Crude membrane fractions from the transfected cells are solubilized in detergent, and the receptors are isolated by affinity chromatography. The receptors are incubated with subunit-specific antibodies, and the resulting receptor-antibody complexes are imaged by AFM. Receptors with two bound antibodies are identified, and the angles between the antibodies are measured. A frequency distribution of these angles then reveals the structure of the receptor. In the present study, we have used this method to show that whereas the GluA1/GluA2 AMPA receptor assembles with an alternating subunit arrangement, the GluN1/GluN2A NMDA receptor adopts an adjacent arrangement. We conclude that, contrary to the current view, the two types of ionotropic glutamate receptor are built in different ways from their constituent subunits.

## EXPERIMENTAL PROCEDURES

### 

#### 

##### Constructs

The following constructs were used: wild type (WT) rat GluA1, rat GluA2igQ with a His_8_/Myc tag between residues 22 and 23 (*i.e.* …FGV^22^HHHHHHHHEQKLISEEDLS^23^SN … ; tag underlined), WT rat GluN1-1a, GluN1 with a hemagglutinin (HA)/His_8_ tag between residues 416 and 417 in the ABD (*i.e.* …TMS^416^YPYDVPDYAHHHHHHHHD^417^GTC … ; tag underlined), GluN1 with a Myc tag between residues 416 and 417 (*i.e.* …TMS^416^EQKLISEEDLD^417^GTC … ; tag underlined), WT rat GluN2A, GluN2A with a FLAG/His_8_ epitope tag between residues 851 and 852, that is, 15 residues downstream of the TMD (*i.e.* …CFTG^851^DYKDDDDKHHHHHHHHV^852^CSD … ; tag underlined), and GluN2A with an HA/His_8_ tag between residues 425 and 426 in the ABD (*i.e.* …DPL^425^EQKLISEEDLHHHHHHHHT^426^ETC … ; tag underlined). All constructs were in the vector pcDNA3.1, except the two AMPA receptor constructs, which were in p3αpA (a derivative of pcDNA3).

##### Antibodies

**FIGURE 1.**
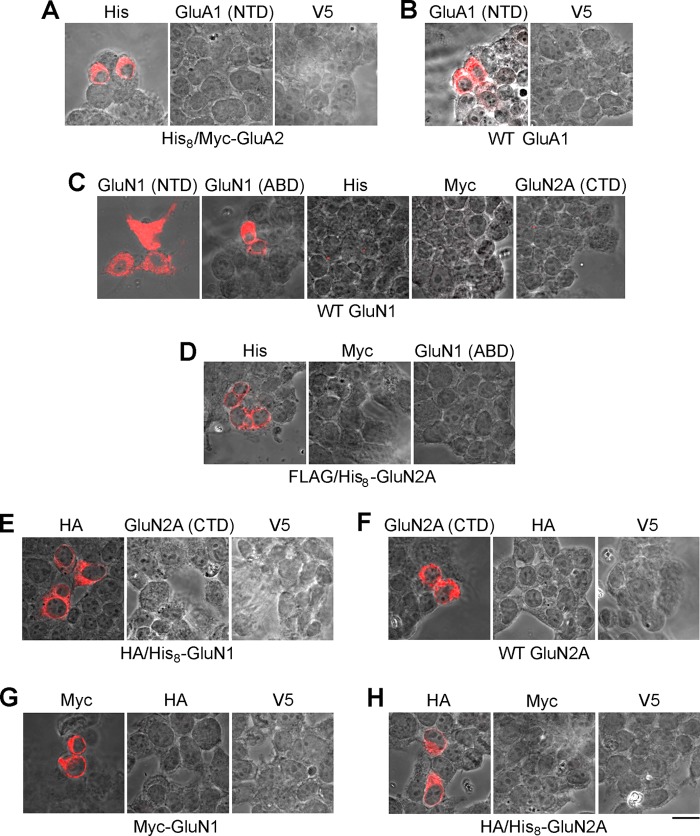
**Demonstration of antibody specificity.** tsA 201 cells expressing His_8_/Myc-GluA2 (*A*), WT GluA1 (*B*), WT GluN1 (*C*), FLAG/His_8_-GluN2A (*D*), HA/His_8_-GluN1 (*E*), WT GluN2A (*F*), Myc-GluN1 (*G*), and HA/His_8_-GluN2A (*H*) were fixed, permeabilized, and probed with monoclonal antibodies to the targets indicated above each *panel*, followed by appropriate Cy3-conjugated goat secondary antibody. Cells were imaged by confocal microscopy, and the fluorescence and brightfield channels were superimposed. *Scale bar*, 50 μm. The relevant figures in the paper are: *A*, Fig. 2; *B*, Figs. 3 and 7; *C*, Figs. 4 and 8; *D*, Figs. 5 and 8; *E–H*, Fig. 6.

The following antibodies were used: mouse monoclonal anti-GluA1 (Millipore; clone RH95, MAB2263, raised against an N-terminal peptide of rat GluA1), mouse monoclonal anti-GluN1 (Abcam; ab134308, S308-48, raised against amino acids 42–361 of GluN1), mouse monoclonal anti-GluN1 (Millipore; clone 54.1, MAB363, raised against amino acids 660–811 of GluN1), rabbit monoclonal anti-GluN2A (Millipore; clone A12W, 04-901, raised against residues 1265–1464 of mouse GluN2A), mouse monoclonal anti-Myc (Invitrogen; R950-25), mouse monoclonal anti-His (Fitzgerald; clone His-17, 10R-P134a), rabbit polyclonal anti-His (Fitzgerald; 70R-HR005), mouse monoclonal anti-V5 (Invitrogen; R960-25), mouse monoclonal anti-HA (Covance; HA.11 clone 16B12, MMS-101P), mouse monoclonal anti-FLAG (Sigma; clone M2, F3165), mouse monoclonal anti-β-actin (Sigma; clone AC-15, A5441), Cy3-conjugated goat anti-mouse (Sigma; C2181), Cy3-conjugated goat anti-rabbit (Sigma; C2306), and fluorescein isothiocyanate-conjugated goat anti-mouse (Sigma; F8771). The specificity of all primary antibodies used to decorate the various AMPA and NMDA receptor subunits was checked by immunofluorescence of suitably transfected cells (Fig. 1).

##### Transient Transfection of tsA 201 Cells

tsA 201 cells were grown in Dulbecco's modified Eagle's medium (Sigma) supplemented with 10% (v/v) fetal bovine serum, 100 units/ml penicillin, and 100 μg/ml streptomycin in an atmosphere of 5% CO_2_/air. Transfection was carried out using the calcium phosphate precipitation method. A total of 250 μg of DNA was used to transfect cells in 5 × 162-cm^2^ culture flasks. For co-transfections, equal amounts of DNA for each construct were used, up to a total of 250 μg. After transfection, cells were incubated for 48 h at 37 °C to allow protein expression. For cells expressing NMDA receptors, 2-amino-5-phosphonovaleric acid (1 μm), 5,7-dichlorokynurenic acid (1 μm), and MK801 (2 μm) were added to the medium to prevent cell toxicity. Protein expression and intracellular localization were checked using immunofluorescence analysis of small scale cultures. Cells were fixed, permeabilized, and incubated with appropriate primary antibodies, followed by fluorophore-conjugated secondary antibodies (Sigma). Cells were imaged by confocal laser scanning microscopy.

##### Isolation of Epitope-tagged Receptors by Immunoaffinity Chromatography

All isolation steps were carried out at 4 °C. Transfected cells were solubilized in a buffer containing 1% (v/v) Triton X-100, 25 mm Tris-HCl, pH 7.5, 150 mm NaCl, 10 mm EDTA, 1 mm PMSF, and protease inhibitor mixture (Complete; Roche Applied Science) for 1 h, before centrifugation at 61,740 × *g* to remove insoluble material. The solubilized extracts were incubated with appropriate antibody-conjugated agarose beads (Sigma) for 3 h. The beads were washed extensively with the above buffer without protease inhibitors, and bound proteins were eluted with appropriate peptide (100 μg/ml in the same buffer). Samples were analyzed by SDS-polyacrylamide gel electrophoresis (PAGE), followed by silver staining and/or immunoblotting, using appropriate primary antibodies, followed by horseradish peroxidase-conjugated goat anti-mouse or anti-rabbit antibodies (Bio-Rad). Immunopositive bands were visualized using enhanced chemiluminescence.

##### Isolation of His_8_-tagged NMDA Receptors by Ni^2+^ Affinity Chromatography

All isolation steps were carried out at 4 °C. Cells were harvested in HEPES-buffered saline (HBS; 100 mm NaCl, 50 mm HEPES, pH 7.6), containing EDTA-free protease inhibitor mixture (Roche Applied Science). Cells were resuspended in lysis buffer (10 mm Tris-HCl, pH 7.6, 2 mm EDTA, containing protease inhibitor mixture) and lysed for 20 min before homogenization with a tight fitting Dounce homogenizer. The homogenate was centrifuged at 500 × *g* for 5 min, and the resulting supernatant was again centrifuged at 21,000 × *g* for 15 min. The pellet was resuspended in solubilization buffer (1% (w/v) CHAPS, 100 mm HEPES, pH 7.6, 0.5 m NaCl, 1 mm PMSF, and protease inhibitor mixture) at a protein concentration of 0.5–1.0 mg/ml. The suspension was agitated at 4 °C for 1 h and then centrifuged at 100,000 × *g* at 4 °C for 1 h to pellet unsolubilized material. Solubilized protein was incubated with Ni^2+^-agarose beads (1 ml of 50% slurry; Probond, Invitrogen) for 30 min. The beads were washed extensively, and protein was eluted with increasing concentrations of imidazole: 2 × 80 mm, 2 × 160 mm, and 2 × 400 mm in wash buffer (0.5-ml fractions). Samples were analyzed by SDS-PAGE, and protein was detected by immunoblotting, as described above. Typically, the first 160 mm fraction was chosen for AFM imaging experiments.

##### Integration of FLAG/His_8_-tagged NMDA Receptors into Liposomes

A crude membrane fraction prepared from transfected cells as described above was solubilized in 40 mm *n*-dodecyl-β-d-maltoside for 1 h. The extract was centrifuged at 60,000 × *g* to remove insoluble material, and the supernatant was subjected to Ni^2+^ affinity chromatography, as described above. The eluted protein sample (usually the second 80 mm and both 160 mm fractions) was concentrated 10-fold using a centrifugal filter (Amicon) and then incubated with anti-FLAG-agarose beads followed by elution with 3×FLAG peptide, as described above. The sample was diluted 5-fold with 1% (w/v) CHAPS and then concentrated using an Amicon filter. Purified proteins were analyzed by SDS-PAGE and immunoblotting, using mouse monoclonal anti-GluN1 and rabbit monoclonal anti-GluN2A antibodies.

Chloroform solutions of 1,2-dioleoyl-*sn*-glycero-3-phosphatidylcholine (Avanti Polar Lipids) and brain l-α-phosphatidylserine (Avanti) were mixed at a molar ratio of 3:1. The chloroform was then evaporated under a stream of nitrogen gas, and the lipids were redissolved in HBS containing 1% (w/v) CHAPS, at a concentration of 2 mg/ml. Solubilized lipid (100 μl; 200 μg) was mixed with 0.5–1 μg of purified NMDA receptor (100 μl) and then dialyzed against detergent-free HBS at room temperature for 3 days. The dialyzed sample was incubated for 12 h at 4 °C with anti-GluN1 or anti-HA (control) monoclonal antibodies and then imaged by AFM.

##### Isolation of Cell Surface Receptors

Intact co-transfected cells were biotinylated by incubation with sulfo-NHS-LC-biotin (Pierce; 1 mg/ml) for 30 min. A Triton X-100 extract of the cells was then produced as described above. Biotinylated proteins were incubated with monomeric avidin-agarose (Pierce) for 1 h. The beads were washed extensively, and bound proteins were eluted with free biotin (2 mm). Eluted receptor was captured using antibody-agarose beads and eluted with appropriate peptide (Sigma), as described above.

##### AFM Imaging in Air

Isolated protein samples were diluted to a final concentration of 0.04 nm, and 45 μl of the sample was allowed to adsorb to freshly cleaved mica disks. After a 5-min incubation, the sample was washed with Biotechnology Performance Certified-grade water (Sigma) and dried under nitrogen. Imaging was performed with a Veeco Digital Instruments Multimode AFM controlled by a Nanoscope IIIa controller. Samples were imaged in air, using tapping mode. The silicon cantilevers used had a drive frequency of ∼300 kHz and a specified spring constant of 40 newtons/m (Olympus). The applied imaging force was kept as low as possible (*A*_s_/*A*_0_ ∼0.85).

For particles within complexes, particle heights and diameters were measured manually using the Nanoscope software and used to calculate molecular volumes, according to Equation 1,

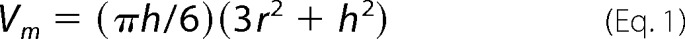
 where *h* is the particle height and *r* is the radius (14). This equation assumes that the adsorbed particles adopt the form of a spherical cap.

Molecular volume based on molecular mass was calculated using Equation 2,

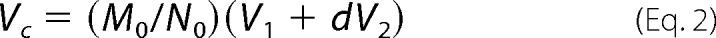
 where *M*_0_ is the molecular mass, *N*_0_ is Avogadro's number, *V*_1_ and *V*_2_ are the partial specific volumes of particle (0.74 cm^3^/g) and water (1 cm^3^/g), respectively, and *d* is the extent of protein hydration (taken as 0.4 g of water/g of protein).

##### AFM Imaging under Fluid

Fluid imaging was carried out using an Olympus fast-scan instrument, as described previously (15). Silicon nitride cantilevers (BL-AC7EGS-A2; Olympus, Tokyo, Japan) with a resonant frequency of 600–1000 kHz in water and a spring constant of 0.1–0.2 newton/m were used. A sharp probe was deposited on each cantilever by electron beam deposition using a Nanotools device (Munich, Germany). A typical free oscillation amplitude was 4 nm, and the amplitude set-point during scanning was ∼70% of this value. A droplet (3 μl) of proteoliposome suspension was deposited on the surface of a freshly cleaved mica disk (diameter 1 mm). After incubation for 30 min at room temperature, the mica surface was gently washed several times with HBS to remove unadsorbed proteoliposomes. AFM imaging in tapping mode was performed in the same buffer solution. AFM images were obtained with a scanning speed of 1 frame/s.

##### Selection of Binding Events

Several criteria were used to identify complexes. Heights and radii were measured for all particles, and the particle volumes were calculated. To be accepted, bound antibodies needed to have a molecular volume between 100 and 300 nm^3^. A cross-section was drawn through the junction between the peripheral and the central particle, and the height of the lowest point between the two particles was measured. This height needed to be >0.3 nm for the peripheral particle to be considered bound. Any particle was rejected if its length was greater than twice its width. To be considered a double binding event, all particles and both binding events needed to meet all of the above criteria.

When proteins are dried down, they flatten extensively on the mica substrate, likely because of electrostatic attractions between the protein and the mica. Nevertheless, it has been shown previously (14) that the molecular volumes of proteins measured by imaging in air are similar to the values obtained by imaging under fluid; hence, the process of drying does not significantly affect the measured molecular volume. It has also been shown by us (16) and by others (14) that there is a close correspondence between the measured and predicted molecular volumes for various proteins over a wide range of molecular masses; hence, molecular volume is measured reasonably accurately by AFM imaging. Furthermore, despite the flattening of the particles, the subunit arrangement within the receptor is faithfully reported by the geometry of the receptor-antibody complexes (11–13).

##### Statistical Analysis

Except for Fig. 4, numerical data in each figure were obtained from single-receptor isolations. In Fig. 4, two separate isolations were performed for both the anti-GluN1 (NTD) decoration and the anti-GluN1 (ABD) decoration.

Histograms were drawn with bin widths chosen according to Scott's equation,

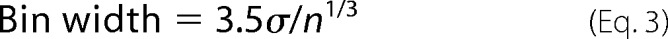
 where σ is an estimate of the standard deviation and *n* is the sample size (17). Where Gaussian curves were fitted to the data, the number of curves was chosen to maximize the *r*^2^ value while giving significantly different means using Welch's *t* test for unequal sample sizes and unequal variances (18).

## RESULTS

**FIGURE 2.**
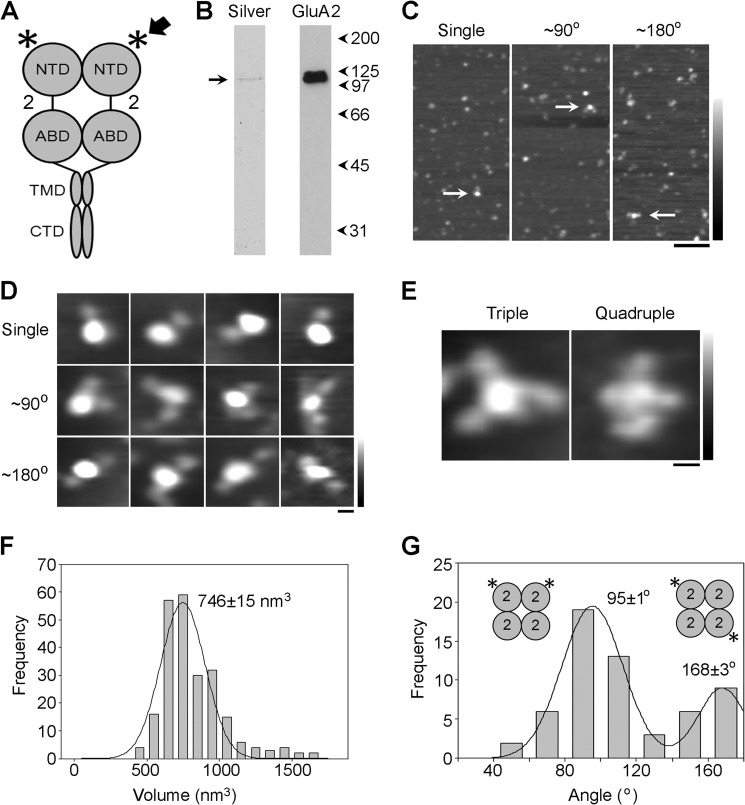
**Subunit arrangement in the total cellular pool of homomeric GluA2 AMPA receptors.** *A*, schematic illustration of a GluA2 AMPA receptor subunit homodimer, with the location of the tag (His_8_/Myc) used to isolate the receptor indicated by the *arrow* and the site of antibody decoration (NTD) indicated by the *asterisk. Numbers* refer to the subunit used (GluA2). *B*, silver-stained gel of the isolated protein (*left*) showing a single major band at a molecular mass of ∼100 kDa (*arrow*). The isolated protein was also analyzed by immunoblotting using an anti-Myc antibody (GluA2, *right*). A single immunopositive band is seen, again at ∼100 kDa. Molecular mass markers (kDa) are shown at the *right. C*, low magnification AFM images of AMPA receptors that had been incubated with anti-His antibodies. *Arrows* indicate single-decorated receptors (*left*) or receptors decorated by two antibodies at either ∼90° (*center*) or ∼180° (*right*). *Scale bar*, 200 nm; *height scale*, 0–4 nm. *D*, gallery of enlarged images showing AMPA receptors after incubation with anti-His antibody. The gallery shows single-decorated receptors (*top*) or receptors decorated by two antibodies at either ∼90° (*middle*) or ∼180° (*bottom*). *Scale bar*, 20 nm; *height scale*, 0–3 nm. *E*, examples of receptors decorated by either three (*left*) or four antibodies (*right*). *Scale bar*, 20 nm; *height scale*, 0–3 nm. *F*, frequency distribution of molecular volumes of the central antibody-decorated particles. The *curve* indicates the fitted Gaussian function. The peak of the distribution (±S.E.) is indicated. *G*, frequency distribution of angles between pairs of bound antibodies. The antibody decoration patterns consistent with data are shown in the *insets*.

tsA 201 cells were initially transfected with DNA encoding GluA2 bearing a His_8_/Myc tag in its NTD. Transfected cells were solubilized in Triton X-100 detergent (1% v/v), and the protein was isolated through the binding of the Myc tag to anti-Myc-agarose beads, followed by elution with a Myc peptide. A schematic illustration of a GluA2 dimer is shown in Fig. 2*A*, with the position of the epitope tag indicated by the *arrow*. A single major band at a molecular mass of ∼100 kDa was seen on both a silver-stained gel and an anti-Myc immunoblot of the isolated protein (Fig. 2*B*).

Isolated AMPA receptors were imaged after incubation with an anti-His antibody, which should decorate the NTD of each subunit (Fig. 2*A*, *asterisk*). Low magnification AFM images of the resulting sample (Fig. 2*C*) showed particles of various sizes, which likely represent receptors in various assembly states (*i.e.* monomers, dimers, and tetramers), as well as uncomplexed antibodies. A population of relatively large particles was seen, some of which were decorated by either one (*arrow*, *left panel*) or two (*arrows*, *center*, and *right panels*) smaller particles (antibodies). The angles between pairs of bound antibodies were either approximately 90° (*center panel*) or approximately 180° (*right panel*). A gallery of single- and double-decorated large particles (at both ∼90° and ∼180°) is shown in Fig. 2*D*. Rarely, triple- and quadruple-decorated large particles were also seen (4 of 929 particles and 2 of 929 particles, respectively); examples are shown in Fig. 2*E*. A frequency distribution of volumes of the decorated central particles, calculated using Equation 1 (Fig. 2*F*), had a single peak at 746 ± 15 nm^3^ (S.E., *n* = 234), close to the expected volume of 760 nm^3^ for an AMPA receptor tetramer, calculated using Equation 2.

Angles between pairs of antibodies bound to the AMPA receptors were measured. A frequency distribution of angles (Fig. 2*G*) had two peaks: a large peak at 95 ± 1° (*n* = 40) and a smaller peak at 168 ± 3° (*n* = 18); the ratio of the numbers of particles in the two peaks was 2.2:1. This angle profile, with two peaks at approximately 90° and 180°, in a ratio of ∼2:1 suggests that the AMPA receptor presents four perpendicular binding sites to the antibody and that these are randomly occupied (Fig. 2*G*, *insets*). The extent of double decoration by a control (anti-V5) antibody was approximately 5-fold lower than that for the anti-GluA1 antibody (Table 1). Similar results were obtained in all other experiments. In addition, when angles for control double decoration events seen in the various experiments were examined, no clear angle peaks could be discerned, indicating that these events were nonspecific.

**TABLE 1:** **Quantitation of antibody decoration of AMPA and NMDA receptors**

Receptors		Antibodies	Single	Double
**AMPA receptor**				
Total	Homomer (Fig. 2)	Anti-His	16.6% (154/929)	5.3% (49/929)
		Anti-V5	7.2% (75/1040)	1.2% (12/1040)
Total	Heteromer (Fig. 3)	Anti-GluA1 (NTD)	24.4% (241/986)	5.0% (62/1248)
		Anti-V5	7.7% (59/768)	0.8% (6/768)
Cell surface	Heteromer (Fig. 7)	Anti-GluA1 (NTD)	18.5% (256/1358)	3.3% (46/1385)
		Anti-V5	8.5% (67/790)	0.6% (5/790)

**NMDA receptor**				
Total	(Fig. 4, *B–F*)	Anti-GluN1 (NTD)	24.2% (109/450)	5.8% (44/762)
		Anti-Myc	5.5% (34/622)	0.9% (7/787)
Total	(Fig. 4, *G–K*)	Anti-GluN1 (ABD)	22.5% (257/1140)	4.1% (47/1140)
		Anti-Myc	5.9% (64/1085)	0.8% (9/1085)
Total	(Fig. 5)	Anti-His	16.4% (125/763)	5.8% (44/763)
		Anti-Myc	7.0% (58/833)	1.3% (11/833)
Total	(Fig. 6, *A–D*)	Anti-HA	18.6% (27/145)	30.3% (44/145)
		Anti-GluN2A (CTD)	23.5% (54/230)	16.1% (37/230)
		Anti-V5	3.8% (1/26)	3.8% (1/26)
Total	(Fig. 6, *E–H*)	Anti-Myc	26.2% (82/313)	21.4% (67/313)
		Anti-HA	31.4% (59/188)	20.7% (39/188)
		Anti-V5	7.7% (6/78)	2.6% (2/78)
Cell surface	(Fig. 8)	Anti-GluN1 (ABD)	16.6% (204/1227)	3.2% (57/1781)
		Anti-His	13.3% (150/1127)	3.6% (51/1429)
		Anti-Myc	5.8% (90/1553)	0.5% (7/1553)

**FIGURE 3.**
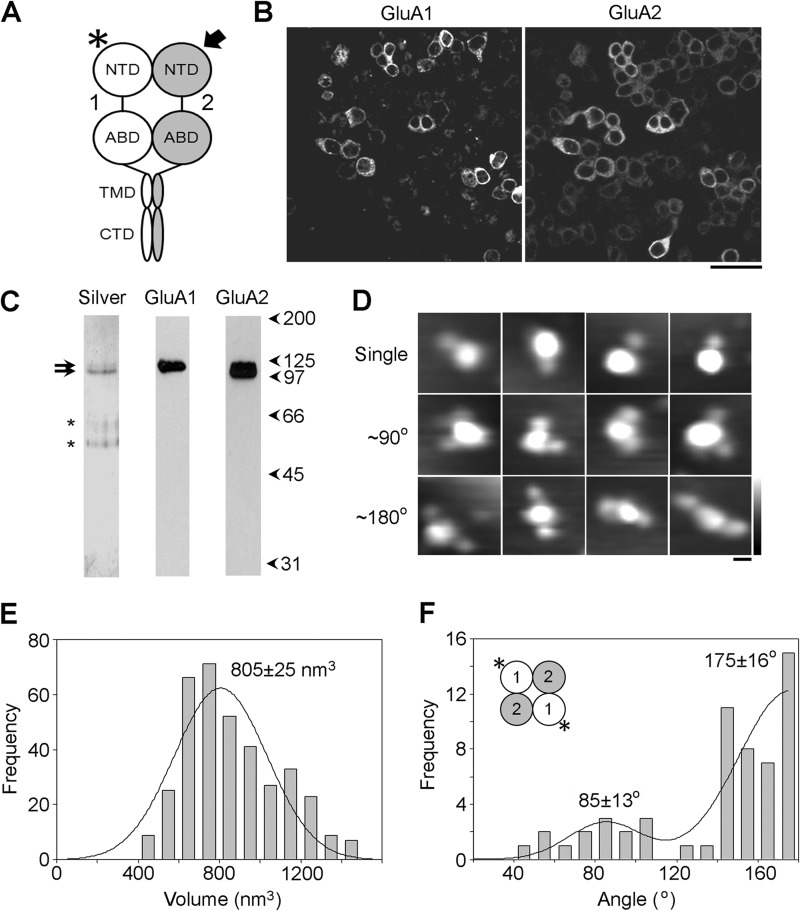
**Subunit arrangement in the total cellular pool of heteromeric GluA1/GluA2 AMPA receptors.** *A*, schematic illustration of a GluA1/GluA2 AMPA receptor subunit heterodimer, with the location of the tag (His_8_/Myc) on GluA2 used to isolate the receptor indicated by the *arrow*, and the site of antibody decoration (NTD of GluA1) indicated by the *asterisk. Numbers* refer to the subunits used (GluA1 and GluA2). *B*, immunofluorescence analysis of protein expression. Cells expressing WT GluA1 plus Myc/His_8_-GluA2 were fixed, permeabilized, and incubated with mouse monoclonal anti-GluA1 and rabbit polyclonal anti-His primary antibodies followed by FITC-conjugated anti-mouse and Cy3-conjugated anti-rabbit secondary antibodies. *Scale bar*, 50 μm. *C*, silver-stained gel of the isolated protein (*left*) showing a doublet at a molecular mass of ∼100 kDa (*arrows*). The *asterisks* indicate bands that appeared even in *blank lanes* and so are likely contaminants in the gel and not in the protein sample. The isolated protein was also analyzed by immunoblotting using anti-GluA1 (*center*) or anti-Myc antibodies (GluA2, *right*). Both antibodies revealed single immunopositive bands at ∼100 kDa. *D*, gallery of enlarged images showing AMPA receptors after incubation with anti-GluA1 antibody. The gallery shows single-decorated receptors (*top*) or receptors decorated by two antibodies at either ∼90° (*center*) or ∼180° (*bottom*). *Scale bar*, 20 nm; *height scale*, 0–3 nm. *E*, frequency distribution of molecular volumes of the central antibody-decorated particles. The curve indicates the fitted Gaussian function. The peak of the distribution is indicated. *F*, frequency distribution of angles between pairs of bound antibodies. The predominant subunit arrangement revealed by the data is shown in the *inset*.

Next, cells were co-transfected with DNA encoding both WT GluA1 and His_8_/Myc-GluA2 (Fig. 3*A*). Immunofluorescence analysis of the transfected cells using anti-GluA1 and anti-His antibodies (Fig. 3*B*) indicated that the majority of the transfected cells expressed both subunits and that a large proportion of the receptors was intracellular. Protein was isolated through the binding of the Myc tag on GluA2 (Fig. 3*A*, *arrow*) to anti-Myc-agarose beads, followed by elution with a Myc peptide. A silver-stained gel of the isolated protein (Fig. 3*C*) showed a doublet of bands at a molecular mass of ∼100 kDa. On the basis of their relatives sizes, we conclude that the smaller, stronger band represents the directly isolated GluA2, whereas the larger (by 3–4 kDa), fainter band represents the indirectly isolated GluA1. The isolated protein was also analyzed by immunoblotting using anti-GluN1 or anti-Myc antibodies (Fig. 3*C*). Both antibodies revealed single immunopositive bands at ∼100 kDa.

The isolated AMPA receptors were imaged after incubation with an anti-GluA1 antibody, which recognizes the NTD (Fig. 3*A*, *asterisk*). Note that the receptor was isolated via GluA2 whereas the antibody was directed against GluA1, thereby ensuring that only heteromers would be decorated. A gallery of single- and double-decorated large particles (at both ∼90° and ∼180°) is shown in Fig. 3*D*. The volume distribution of decorated central particles (Fig. 3*E*) had a single peak at 805 ± 25 nm^3^ (*n* = 363), close to the volume of the GluA2 receptor homomer (746 nm^3^; Fig. 2*F*). The distribution of the angles between pairs of bound antibodies (Fig. 3*F*) had two peaks: a large peak at 175 ± 16° (*n* = 43) and a smaller peak at 85 ± 13° (*n* = 14). This angle profile suggests that in most of the receptors, the decorated GluA1 subunits are diagonally opposite each other and that the predominant subunit arrangement is therefore 1/2/1/2 (Fig. 3*F*, *inset*). The presence of a small peak at ∼90° suggests that a minority (∼25%) of the receptors adopt the opposite arrangement of 1/1/2/2.

**FIGURE 4.**
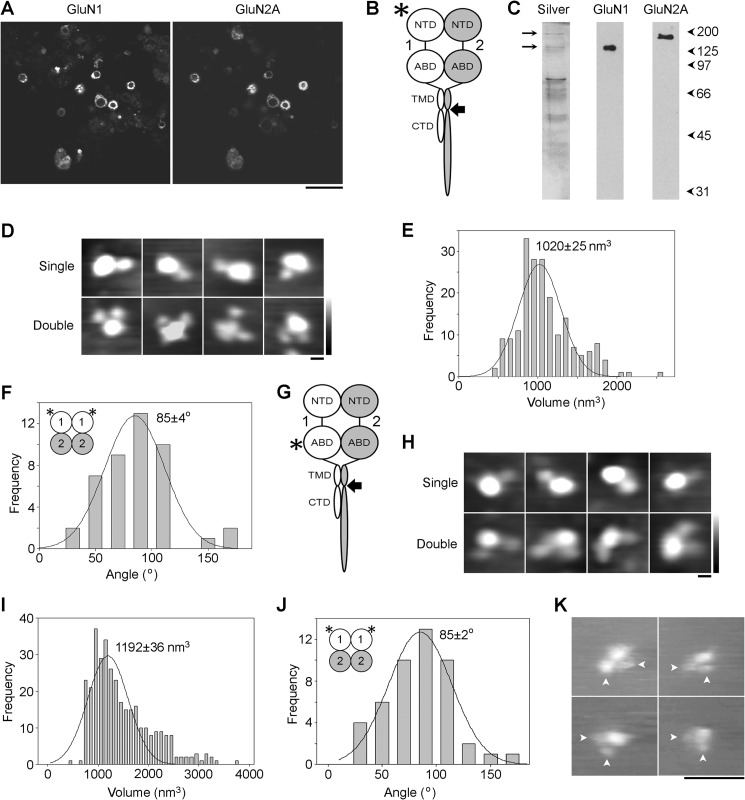
**Arrangement of GluN1 subunits in GluN1/GluN2A NMDA receptors isolated from the total cellular pool.** *A*, immunofluorescence analysis of protein expression. Cells expressing WT GluN1 plus FLAG/His_8_-GluN2A were fixed, permeabilized, and incubated with mouse monoclonal anti-GluN1 (ABD) and rabbit monoclonal anti-GluN2A primary antibodies followed by FITC-conjugated anti-mouse and Cy3-conjugated anti-rabbit secondary antibodies. *Scale bar*, 50 μm. *B*, schematic illustration of a GluN1/GluN2A NMDA receptor subunit heterodimer, with the location of the tag (FLAG/His_8_) used to isolate the receptor indicated by the *arrow*, and the site of anti-GluN1 antibody decoration (NTD) indicated by the *asterisk. Numbers* refer to the subunits used (GluN1 and GluN2A). *C*, silver-stained gel of the isolated protein (*left*) showing the presence of bands at ∼120 and ∼180 kDa (*arrows*). Immunoblotting using anti-subunit antibodies indicated that these two bands were GluN1 and GluN2A, respectively (*center* and *right*). *D*, gallery of enlarged images showing receptors after incubation with anti-GluN1 (NTD) antibody. The gallery shows single- (*upper*) and double-decorated receptors (*lower*). *Scale bar*, 20 nm; *height scale*, 0–3 nm. *E*, frequency distribution of molecular volumes of the central particles decorated by anti-GluN1 (NTD) antibodies. The curve indicates the fitted Gaussian function. The peak of the distribution is indicated. *F*, frequency distribution of angles between pairs of bound antibodies. The subunit arrangement revealed by the data is shown in the *inset. G*, schematic illustration of a GluN1/GluN2A NMDA receptor subunit heterodimer, with the location of the tag (FLAG/His_8_) used to isolate the receptor indicated by the *arrow*, and the site of anti-GluN1 antibody decoration (ABD) indicated by the *asterisk. H*, gallery of enlarged images showing receptors after incubation with anti-GluN1 (ABD) antibody. The gallery shows single- (*upper*) and double-decorated receptors (*lower*). *Scale bar*, 20 nm; *height scale*, 0–3 nm. *I*, frequency distribution of molecular volumes of the central particles decorated by anti-GluN1 (ABD) antibodies. *J*, frequency distribution of angles between pairs of bound antibodies. The subunit arrangement revealed by the data is shown in the *inset. K*, gallery of images of double-decorated receptors integrated into supported lipid bilayers and imaged under fluid. Bound antibodies are indicated by the *arrowheads. Scale bar*, 100 nm; *height range*, 20 nm.

When cells were co-transfected with DNA encoding WT GluN1 and GluN2A with a FLAG/His_8_ tag just downstream of its TMD, immunofluorescence analysis of the transfected cells using anti-GluN1 and anti-GluN2A antibodies (Fig. 4*A*) indicated that the majority of the transfected cells expressed both subunits. GluN1/GluN2A NMDA receptors were routinely isolated by anti-FLAG affinity chromatography (Fig. 4*B*, *arrow*). Note that we have shown previously (15) that NMDA receptors containing tagged GluN2A are delivered efficiently to the plasma membrane of *Xenopus laevis* oocytes and function normally. Analysis of a typical isolated sample on a silver-stained gel revealed the presence of bands at ∼120 and ∼180 kDa (Fig. 4*C*, *arrows*). Immunoblotting using appropriate anti-subunit antibodies indicated that these two bands were GluN1 and GluN2A, respectively. Note that contaminant bands were also seen on the silver-stained gel; however, these would not become decorated by antibodies and would therefore be excluded from the subsequent analysis of subunit arrangement.

The NMDA receptors were first imaged after incubation with an anti-GluN1 antibody that recognizes a region within the NTD (Fig. 4*B*, *asterisk*). A gallery of single- and double-decorated receptors is shown in Fig. 4*D*. The volume distribution of decorated central particles (Fig. 4*E;* data from two separate preparations) had a single peak at 1020 ± 25 nm^3^ (*n* = 196), close to the expected volume of 1140 nm^3^ for a GluN1/GluN2A NMDA receptor of molecular mass ∼600 kDa (assuming a 2:2 subunit stoichiometry). The distribution of angles between pairs of bound antibodies (Fig. 4*F*) had a single peak at 85 ± 4° (*n* = 44). This angle profile suggests that in the GluN1/GluN2A NMDA receptor the decorated GluN1 subunits are adjacent and that the subunit arrangement is therefore 1/1/2/2 (Fig. 4*F*, *inset*). This arrangement is in stark contrast to the predominant 1/2/1/2 arrangement in the GluA1/GluA2 AMPA receptor (above).

NMDA receptors were also imaged after incubation with an anti-GluN1 antibody that recognizes a region within the ABD (Fig. 4*G*, *asterisk*). A gallery of single- and double-decorated receptors is shown in Fig. 4*H*. The volume distribution of decorated central particles (Fig. 4*I*; data from two separate preparations) had a single peak at 1192 ± 36 nm^3^ (*n* = 339), close to the expected volume of 1140 nm^3^. The distribution of angles between pairs of bound antibodies (Fig. 4*J*) had a single peak at 85 ± 2° (*n* = 47), consistent with the 1/1/2/2 subunit arrangement suggested above (Fig. 4*J*, *inset*).

We have shown previously that AFM imaging of antibody-tagged receptors in air reliably reveals the receptor subunit arrangement (11–13). Nevertheless, to exclude the possibility of artifacts introduced by drying the receptor onto the mica supports, the GluN1/GluN2A receptor was also imaged in its native state (*i.e.* under fluid) after integration into a supported lipid bilayer. Fig. 4*K* shows a gallery of four receptors each of which was decorated by two antibodies (*arrowheads*) directed against the ABD of the GluN1 subunit. The mean angle between the pairs of antibodies was 78 ± 5° (*n* = 4), consistent with the 1/1/2/2 subunit arrangement indicated by the dry images. No decorated particles were seen when a control antibody (anti-HA) was used.

**FIGURE 5.**
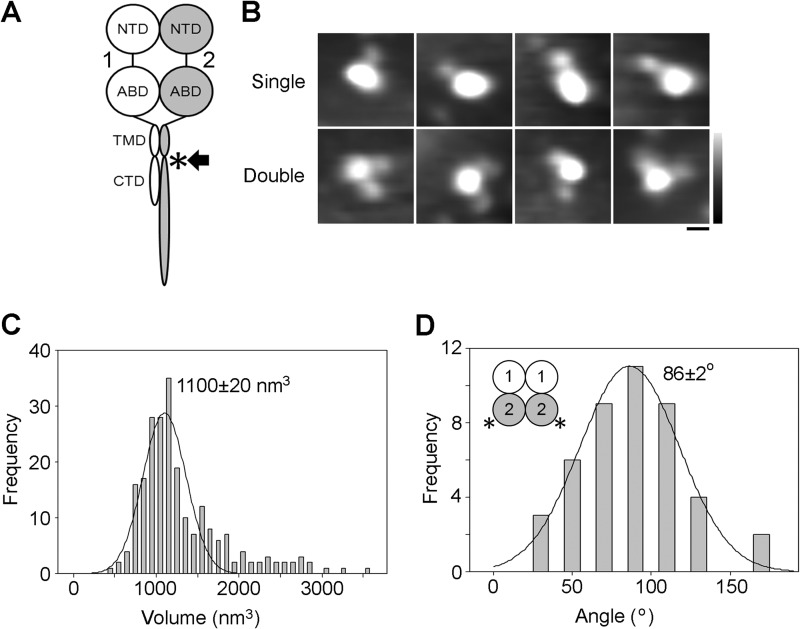
**Arrangement of GluN2A subunits in GluN1/GluN2A NMDA receptors isolated from the total cellular pool.** *A*, schematic illustration of a GluN1/GluN2A NMDA receptor subunit heterodimer, with the location of the tag (FLAG/His_8_) used to isolate the receptor indicated by the *arrow*, and the site of anti-His antibody decoration (post-TMD) indicated by the *asterisk. Numbers* refer to the subunits used (GluN1 and GluN2A). *B*, gallery of enlarged images showing receptors after incubation with anti-His antibody. The gallery shows single- (*upper*) and double-decorated receptors (*lower*). *Scale bar*, 20 nm; *height scale*, 0–3 nm. *C*, frequency distribution of molecular volumes of the central particles decorated by anti-His antibodies. The curve indicates the fitted Gaussian function. The peak of the distribution is indicated. *D*, frequency distribution of angles between pairs of bound antibodies. The subunit arrangement revealed by the data is shown in the *inset*.

Results very similar to those described above were obtained when the same GluN1/GluN2A NMDA receptor, isolated in a separate experiment by anti-FLAG immunoaffinity chromatography, was incubated with an anti-His antibody, which will recognize the His_8_ tag on GluN2A (Fig. 5*A*, *asterisk*). A gallery of single- and double-decorated receptors is shown in Fig. 5*B*. In this experiment, the volume distribution of decorated central particles had a single peak at 1100 ± 20 nm^3^ (*n* = 224; Fig. 5*C*), and the angle distribution had a single peak at 86 ± 2° (*n* = 44; Fig. 5*D*), again indicating a 1/1/2/2 subunit arrangement (Fig. 5*D*, *inset*).

**FIGURE 6.**
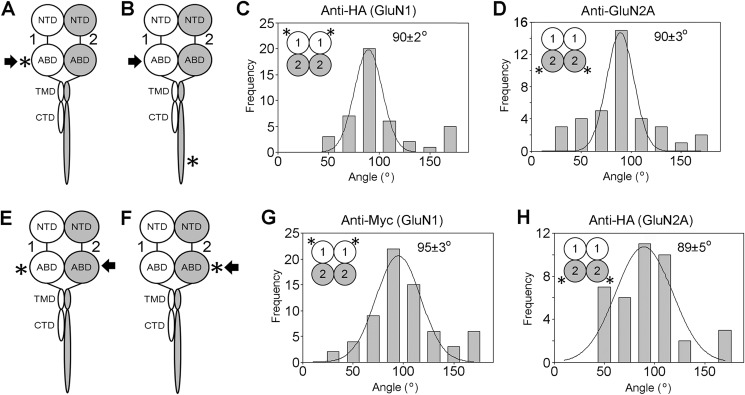
**Antibody decoration of additional epitope-tagged NMDA receptors isolated from the total cellular pool.** *A* and *B*, schematic illustration of a GluN1/GluN2A NMDA receptor subunit heterodimer, with the location of the tag (HA/His_8_) used to isolate the receptor indicated by the *arrow*, and the sites of antibody decoration (*A*, anti-HA (ABD), and *B*, anti-GluN2A (CTD)) indicated by the *asterisk. Numbers* refer to the subunits used (GluN1 and GluN2A). *C*, frequency distribution of angles between pairs of bound anti-HA antibodies. The *curve* indicates the fitted Gaussian function. The peak of the distribution is indicated. *D*, frequency distribution of angles between pairs of bound anti-GluN2A antibodies. *E* and *F*, schematic illustration of a GluN1/GluN2A NMDA receptor subunit heterodimer, with the location of the tag (HA/His_8_) used to isolate the receptor indicated by the *arrow*, and the sites of antibody decoration (*E*, anti-Myc (ABD), and *F*, anti-HA (ABD)) indicated by the *asterisk. G*, frequency distribution of angles between pairs of bound anti-Myc antibodies. *H*, frequency distribution of angles between pairs of bound anti-HA antibodies. For each distribution, the subunit arrangement revealed by the data is shown in the *inset*.

We also investigated two other combinations of GluN1 and GluN2A subunits tagged at different sites. Data for these subunit combinations are shown in Fig. 6. GluN1 tagged in an ABD loop by HA/His_8_ was co-expressed with WT GluN2A, and the receptor was isolated through binding of the His_8_ tag on GluN1 (Fig. 6, *A* and *B*, *arrows*) to Ni^2+^-agarose, followed by elution with imidazole. The receptors were decorated by either an anti-HA antibody (Fig. 6*A*, *asterisk*) or an anti-GluN2A antibody that recognizes the CTD (Fig. 6*B*, *asterisk*); in both cases the frequency distributions of angles between pairs of bound antibodies had single peaks at 90° (Fig. 6, *C* and *D*). In addition, GluN1 tagged in an ABD loop by Myc was co-expressed with GluN2A tagged in an ABD loop by HA/His_8_, and the receptor was isolated via the His_8_ tag on GluN2A (Fig. 6, *E* and *F*, *arrows*). The receptors were decorated by either anti-Myc (Fig. 6*E*, *asterisk*) or anti-HA (Fig. 6*F*, *asterisk*) antibodies; once again, the frequency distributions of angles between pairs of bound antibodies had single peaks close to 90° (Fig. 6, *G* and *H*). These additional results also point to a 1/1/2/2 subunit arrangement in the GluN1/GluN2A NMDA receptor (Fig. 6, *C*, *D*, *G*, and *H*, *insets*).

It is clear from our immunofluorescence analysis that large proportions of both AMPA and NMDA receptors expressed in the tsA 201 cells are intracellular. Because it is known that ionotropic glutamate receptors undergo structural rearrangements en route to the plasma membrane (10, 19), we devised a method for isolating cell surface receptors. Specifically, cells were biotinylated using the membrane-impermeant biotinylation reagent sulfo-NHS-LC-biotin. Cells were then solubilized in Triton X-100 detergent, and biotinylated proteins were captured on monomeric avidin-agarose beads before elution with free biotin. Eluted proteins were then subjected to the normal immunoaffinity purification process. Using this method we could be sure that the proteins being analyzed had reached the cell surface.

**FIGURE 7.**
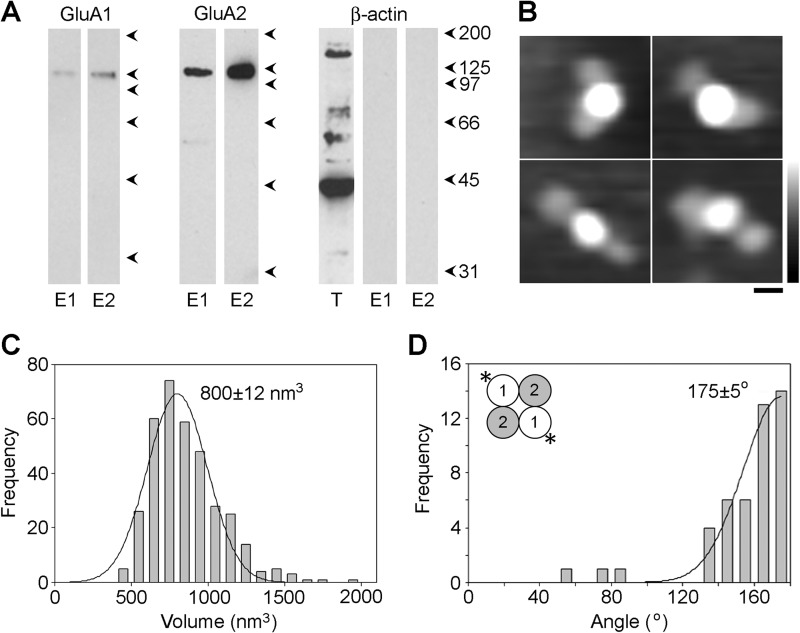
**Subunit arrangement in cell surface GluA1/GluA2 AMPA receptors.** *A*, protein eluted from monomeric avidin-agarose by biotin (*E1*) and from anti-Myc-agarose by Myc peptide (*E2*) was analyzed by immunoblotting using anti-GluA1 (*left*), anti-Myc (GluA2, *center*), or anti-β-actin antibodies (*right*). The *right panel* also shows an immunoblot of the total cell extract (*T*). *B*, gallery of enlarged images showing receptors double decorated by anti-GluA1 (NTD) antibodies. *Scale bar*, 20 nm; *height scale*, 0–3 nm. *C*, frequency distribution of molecular volumes of the central antibody-decorated particles. The curve indicates the fitted Gaussian function. The peak of the distribution is indicated. *D*, frequency distribution of angles between pairs of bound antibodies. The subunit arrangement revealed by the data is shown in the *inset*.

Cells were transfected with DNA encoding both WT GluA1 and GluA2 with a His_8_/Myc tag in the NTD. Protein eluted from avidin beads by biotin and subsequently from anti-Myc beads by Myc peptide, gave clear, positive signals on immunoblots with both anti-GluA1 and anti-Myc antibodies (Fig. 7*A*). In contrast, blots of the same samples with an anti-β-actin antibody were negative, indicating that no intracellular protein was captured on the beads.

The isolated cell surface AMPA receptors were imaged after incubation with anti-GluA1 antibody (Fig. 7*B*). The volume distribution of decorated central particles (Fig. 7*C*) had a single peak at 800 ± 12 nm^3^ (*n* = 351), close to the volumes of receptors isolated from total cell extracts. In contrast to the result for total cellular GluA1/GluA2 receptor, the distribution of the angles between pairs of bound antibodies (Fig. 7*D*) now had only one peak, at 175 ± 5° (*n* = 46), with only three decoration events at <100°. This result supports the above conclusion that the subunit arrangement in the AMPA receptor heteromer is 1/2/1/2 and indicates that the small peak at ∼90° seen for the total cell extract likely represents an intracellular pool of “incorrectly ” assembled receptors with a 1/1/2/2 subunit arrangement.

**FIGURE 8.**
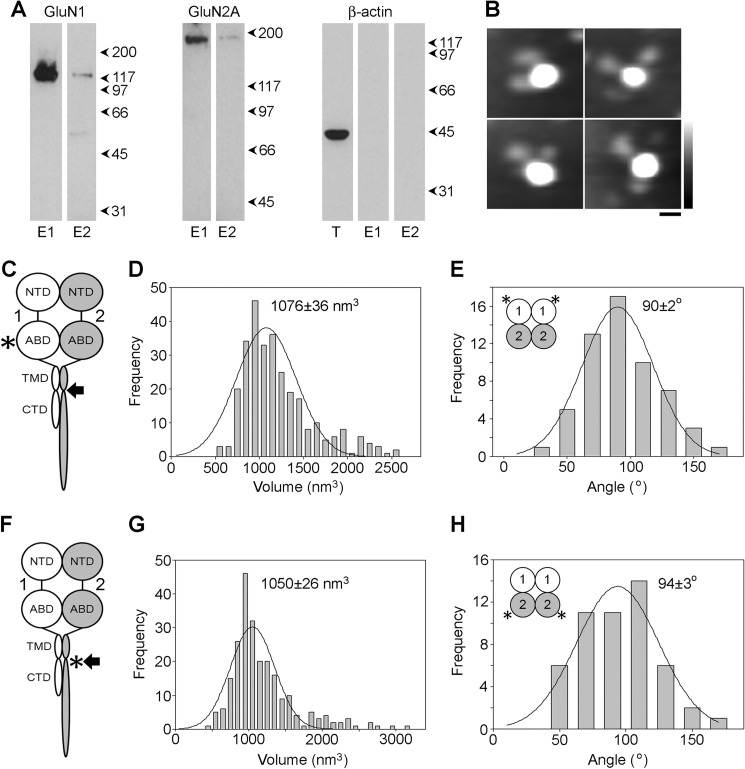
**Subunit arrangement in cell surface GluN1/GluN2A NMDA receptors.** *A*, protein eluted from monomeric avidin-agarose by biotin (*E1*) and from anti-FLAG-agarose by triple-FLAG peptide (*E2*) was analyzed by immunoblotting using anti-GluN1 (*left*), anti-GluN2A (*center*), or anti-β-actin antibodies (*right*). The *right panel* also shows an immunoblot of the total cell extract (*T*). *B*, gallery of enlarged images showing receptors double decorated by anti-GluN1 (ABD) antibodies. *Scale bar*, 20 nm; *height scale*, 0–3 nm. *C*, schematic illustration of a GluN1/GluN2A NMDA receptor subunit heterodimer, with the location of the tag (FLAG/His_8_) used to isolate the receptor indicated by the *arrow*, and the site of anti-GluN1 antibody decoration (ABD) indicated by the *asterisk. Numbers* refer to the subunits used (GluN1 and GluN2A). *D*, frequency distribution of molecular volumes of the central particles decorated by anti-GluN1 antibodies. The curve indicates the fitted Gaussian function. The peak of the distribution is indicated. *E*, frequency distribution of angles between pairs of bound antibodies. The subunit arrangement revealed by the data is shown in the *inset. F*, schematic illustration of a GluN1/GluN2A NMDA receptor subunit heterodimer, with the location of the tag used to isolate the receptor indicated by the *arrow*, and the site of anti-His antibody decoration (post-TMD) indicated by the *asterisk. G*, frequency distribution of molecular volumes of the central particles decorated by anti-His antibodies. *H*, frequency distribution of angles between pairs of bound antibodies. The subunit arrangement revealed by the data is shown in the *inset*.

The same procedure as that described for GluA1/GluA2 AMPA receptors was used to isolate cell surface GluN1/GluN2A NMDA receptors. Cells were transfected with DNA encoding both WT GluN1 and FLAG/His_8_-tagged (post-TMD) GluN2A. Protein eluted from avidin beads by biotin and subsequently from anti-FLAG beads by FLAG peptide gave clear, positive signals on immunoblots with both anti-GluN1 and anti-GluN2A antibodies (Fig. 8*A*). In contrast, blots of the same samples with an anti-β-actin antibody were negative, indicating that no intracellular protein was captured on the beads.

The isolated cell surface NMDA receptors were imaged after incubation with an anti-GluN1 antibody (Fig. 8*B*), which recognizes the ABD (Fig. 8*C*, *asterisk*). The volume distribution of decorated central particles (Fig. 8*D*) had a single peak at 1076 ± 36 nm^3^ (*n* = 290), close to the volumes of receptors isolated from total cell extracts. The distribution of angles between pairs of bound antibodies (Fig. 8*E*) had one peak, at 90 ± 2° (*n* = 57). Very similar results were obtained when the GluN1/GluN2A NMDA receptor was incubated with an anti-His antibody, which recognized the post-TMD His_8_ tag on GluN2A (Fig. 8*F*, *asterisk*). In this case, the volume distribution of decorated central particles had a single peak at 1050 ± 26 nm^3^ (*n* = 236; Fig. 8*G*), and the angle distribution had a single peak at 94 ± 3° (*n* = 51; Fig. 8*H*). Taken together, these results demonstrate that GluN1/GluN2A NMDA receptors expressed at the cell surface adopt a 1/1/2/2 subunit arrangement.

**FIGURE 9.**
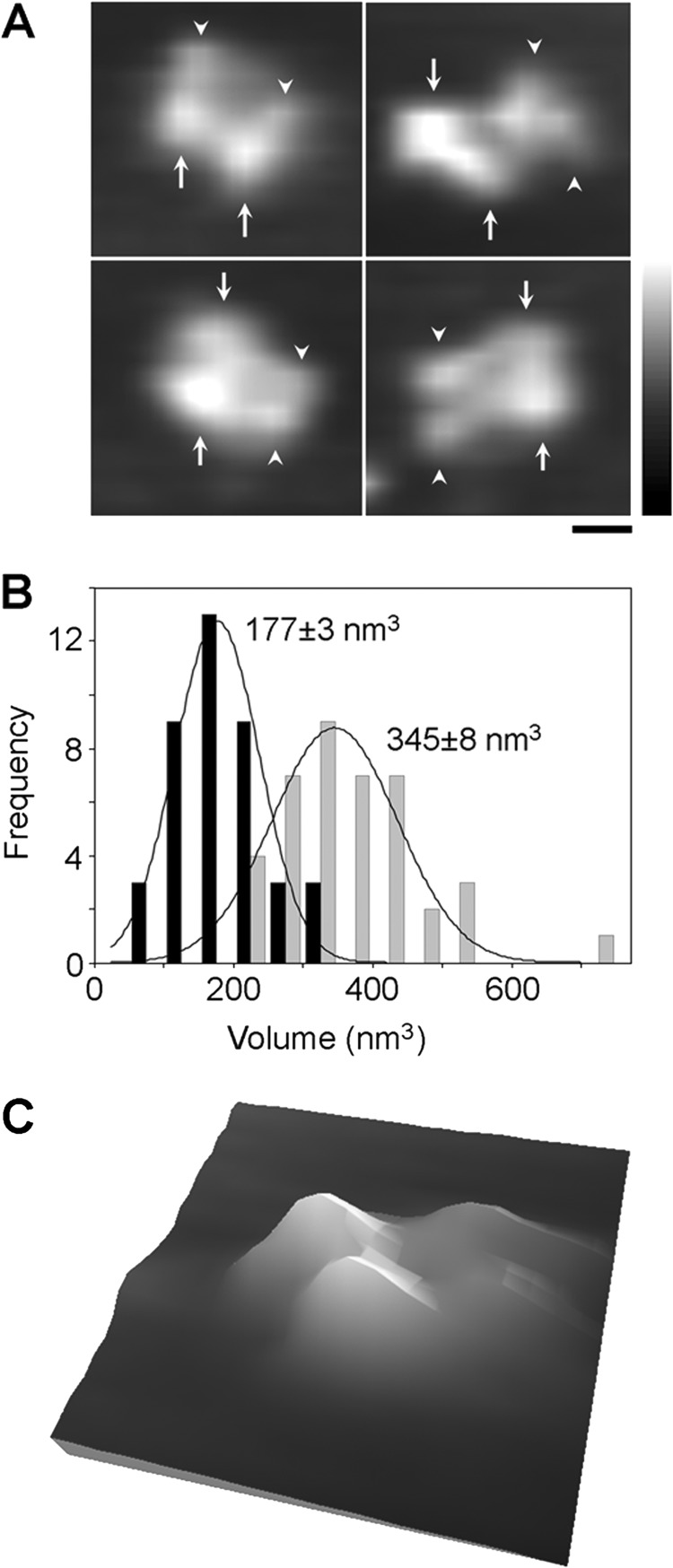
**Structure of partially dissociated GluN1/GluN2A NMDA receptors.** *A*, examples of structures composed of four particles that are likely GluN1/GluN2A receptors that have attached to the mica intact and then partially dissociated. Adjacent small and large particles are indicated by *arrowheads* and *arrows*, respectively. *Scale bar*, 100 nm; *height scale*, 0–3 nm. *B*, frequency distribution of molecular volumes of individual small (*black bars*) and large particles (*gray bars*) within four-particle clusters. The curves indicate the fitted Gaussian functions. The means of the distributions are indicated. *C*, three-dimensional representation of a partially dissociated NMDA receptor. The image is 80 nm square.

Adhesion of individual protein molecules to the mica substrate involves electrostatic forces that are sometimes large enough to partially dissociate protein complexes into their individual subunits (20). Here, we identified a number of structures that consisted of four individual components, two small and two large (Fig. 9*A*). In each case the smaller components (*arrowheads*) and the larger components (*arrows*) were adjacent to each other, in a manner consistent with the 1/1/2/2 subunit arrangement proposed above. The frequency distributions of volumes of the individual small and large particles (Fig. 9*B*) had peaks at 177 ± 3 nm^3^ and 345 ± 8 nm^3^ (*n* = 40), close to the predicted volumes of individual GluN1 and GluN2A subunits (228 nm^3^ and 342 nm^3^, respectively). Hence, these structures are likely to be partially dissociated NMDA receptors, which had assembled with a 1/1/2/2 subunit arrangement. A three-dimensional representation of a partially dissociated NMDA receptor is shown in Fig. 9*C*.

## DISCUSSION

Recently, there have been reports of a 1/2/1/2 subunit arrangement in GluN1/GluN2A NMDA receptors (7–10). Three of these four studies (7, 9, 10) have used intersubunit cross-linking (or copper coordination) by introduced cysteine residues to map out regions of the receptor that are close together. Crucially, as acknowledged in the reports, the deduction of the subunit arrangement from the experimental data relies on the assumption that the NMDA receptor assembles in the same way as the AMPA receptor. Our data, based on extensive structural analysis of intact receptors, decorated at various positions on either GluN1 or GluN2A, strongly suggest that this is not the case and point instead to a 1/1/2/2 arrangement.

In fact, several previous approaches, including electrophysiological analysis of the behavior of combinations of receptor concatemers and fragments (5) and Förster resonance energy transfer (6), have also indicated a 1/1/2/2 subunit arrangement in NMDA receptors. Further, there is considerable evidence that GluN1 dimerization represents an early step in NMDA receptor assembly (19, 21) and that disulfide binding between pairs of GluN1 NTDs is required for expression of the receptor at the cell surface (22).

The NTDs of ionotropic glutamate receptors adopt a clamshell arrangement, composed of two domains, L1 and L2. Interestingly, whereas non-NMDA receptor NTDs form dimers through L1/L1 and L2/L2 interactions (4, 7, 23, 24), the L1 and L2 regions of the NMDA receptor NTD are twisted by approximately 50° relative to each other, so that the NTDs associate with each other through L1/L1 interactions only (19, 25–27). Hence, it is already clear that NMDA and non-NMDA receptors assemble differently. Our results point to more fundamental differences in the structures of NMDA and non-NMDA receptors, necessitating a reappraisal of current ideas about the assembly of ionotropic glutamate receptors.

## Footnotes

4 The abbreviations used are:

NTD: N-terminal domain
ABD: agonist-binding domain
AFM: atomic force microscopy
CTD: C-terminal domain
HBS: HEPES-buffered saline
TMD: transmembrane domain.
